# Spirituality and health-related quality of life in patients with lung cancer

**DOI:** 10.1590/0034-7167-2024-0457

**Published:** 2025-07-11

**Authors:** Erica Maria Belmiro dos Santos, Kátia Neyla de Freitas Macedo Costa, Maria das Graças Melo Fernandes, Lia Raquel de Carvalho Viana, Cleane Rosa Ribeiro da Silva, Maria Cristina Lins Oliveira Frazão, Gerlania Rodrigues Salviano Ferreira, Ana Luísa Fernandes Vieira Melo

**Affiliations:** I Universidade Federal da Paraíba. João Pessoa, Paraíba, Brazil

**Keywords:** Spirituality, Quality of Life, Lung Neoplasms, Oncology Nursing, Comprehensive Health Care., Espiritualidad, Calidad de Vida, Neoplasias Pulmonares, Enfermería Oncológica, Atención Integral de Salud.

## Abstract

**Objective:**

To correlate spirituality with the health-related quality of life (HRQoL) in patients with lung cancer.

**Methods:**

A quantitative study was conducted with 74 patients at an oncology hospital in João Pessoa, Paraíba, from July to December 2023. Interviews were conducted using the World Health Organization Quality of Life Spirituality - Religiosity - Personal Beliefs (WHOQOL-SRPB) and the European Organization for Research and Treatment of Cancer Quality of Life Questionnaire (EORTC QLQ-C30). Data were processed and analyzed using descriptive and inferential statistics.

**Results:**

A weak positive correlation was observed between the connection score and financial difficulties (p = 0.012), as well as a weak to moderate negative correlation between hope and the cognitive score (p = 0.001) on the spirituality and HRQoL scales.

**Conclusion:**

Aspects of spirituality contribute to the improvement of HRQoL in patients, providing a basis for the development of individualized care plans.

## RESUMEN

**Objetivo:**

Correlacionar la espiritualidad con la calidad de vida relacionada con la salud en pacientes con cáncer de pulmón.

**Métodos:**

Estudio con enfoque cuantitativo, realizado con 74 pacientes en un hospital oncológico en João Pessoa, Paraíba, durante el período de julio a diciembre de 2023. Las entrevistas se llevaron a cabo utilizando los instrumentos World Health Organization Quality of Life Spirituality - Religiosity - Personal Beliefs (WHOQOL-SRPB) y European Organization for Research and Treatment of Cancer Care Quality of Life Questionnaire (EORTC QLQ-C30). Los datos fueron procesados y analizados mediante estadística descriptiva e inferencial.

**Resultados:**

Se observó una correlación positiva débil entre la puntuación de conexión y la dificultad financiera (p = 0,012), así como una correlación negativa débil a moderada entre esperanza y la puntuación cognitiva (p = 0,001) en las escalas de espiritualidad y calidad de vida relacionada con la salud.

**Conclusión:**

Las facetas de la espiritualidad contribuyen a mejorar la calidad de vida relacionada con la salud de los pacientes, proporcionando fundamentos para el diseño de un plan de cuidado individualizado.

## INTRODUCTION

Lung cancer cases are increasing globally^(1)^. In the United States, it is the second most common cancer and the leading cause of cancer-related deaths. In developing countries, incidence and prevalence rates continue to rise, driven by smoking, which remains the primary risk factor^(2)^.

In Brazil, an estimated 32,000 new cases of lung cancer are expected between 2023 and 2025, according to data from the National Cancer Institute (INCA in Portuguese) ^(3)^. Lung cancer is classified into small-cell and non-small-cell types for therapeutic and prognostic purposes. Both types are characterized by high lethality, low cure rates, and limited survival^(4)^.

Lung cancer and its treatment are associated with debilitating symptoms such as pain, dyspnea, cough, and fatigue. These symptoms, along with other stressors, can trigger various cognitive and affective responses, including depressive symptoms, anxiety, and negative feelings toward God due to the diagnosis. These psycho-spiritual manifestations interfere with treatment adherence and quality of life (QoL) ^(5)^.

Conversely, researchers have identified that spiritual well-being serves as a protective factor against the suffering caused by the disease, contributing to an overall better QoL^(6-8)^. Spirituality is considered an inseparable aspect of human subjectivity, influencing physical, psychosocial, and cultural well-being. It represents an individual’s search for meaning, purpose, and connection with the sacred^(9)^. Thus, spiritual facets encompass the multidimensionality of the individual and are interconnected with their well-being and health-related quality of life (HRQoL).

According to the World Health Organization (WHO), QoL is defined as an individual’s perception of themselves in the context of their living environment^(10)^. When related to health interventions, it is referred to as HRQoL, which encompasses cognitive, functional, psychological, and social aspects of the individual^(10,11)^. In the context of lung cancer, HRQoL is impacted by various factors related to the disease, leading to physical, emotional, familial, and social challenges^(11)^.

A study conducted in Singapore among patients with lung cancer found that higher levels of psychological and functional needs were associated with poorer HRQoL^(11)^. In this context, spirituality may mitigate HRQoL impairment by offering strategies that allow patients to reinterpret the meaning of life, promoting resilience and determination in the face of the difficulties posed by the disease^(12)^.

Given this scenario, the spiritual dimension should be incorporated into nursing care plans through actions that encourage the development of patients’ beliefs as a means of empowerment. This approach aims to provide support and comfort during the oncological disease experience^(13)^. The hypothesis of this study is that spirituality influences the HRQoL of patients with lung cancer. Thus, the correlation between these two variables underscores the importance of addressing the multidimensionality of individuals with cancer, helping to prevent the negative impacts caused by the disease.

## OBJECTIVE

To correlate spirituality with the health-related quality of life (HRQoL) of patients with lung cancer undergoing oncological treatment.

## METHODS

### Ethical Aspects

The project was approved by the Research Ethics Committee (REC) of the Health Sciences Center (CCS) at the Federal University of Paraíba (UFPB). The study was conducted in accordance with the guidelines set forth in Resolution No. 466/2012 of the National Health Council (CNS) of the Brazilian Ministry of Health (MS). All participants signed the Informed Consent Form (ICF) at the time of data collection. Participation was extended to those who met the inclusion criteria and were informed about the risks and benefits of the study, as well as the assurance of anonymity throughout all stages of the research.

### Study Design, Period, and Location

This was a descriptive and cross-sectional study with a quantitative approach^(14)^, guided by the STROBE tool. It was conducted in a large, high-complexity state reference hospital for oncological treatment located in João Pessoa, Paraíba, Brazil. Data collection occurred through individual interviews conducted in the waiting room for chemotherapy or radiotherapy sessions, between July and December 2023.

### Population and Sample; Inclusion and Exclusion Criteria

The sample selection was based on the number of patients treated by a thoracic surgeon in the reference outpatient service from January to June 2023, totaling 253 patients. Assuming that the population was homogeneous regarding the primary study variables, a sample size was calculated to detect weak, moderate, or strong correlations, with a minimum linear correlation coefficient of 0.32. The significance level was set at 5%, corresponding to a 95% confidence level and a power of 80%. The sample size was calculated using the following formula:


$$n=\left(\frac{Z_{a}+Z_{\beta }}{\frac{1}{2}\log\left(\frac{1+r}{1-r}\right)}\right)+3,$$

Where α represents the significance level, 1 - *β* is the power, *Z* ( . ) is the quantile of the standard normal distribution, and r is the estimated linear correlation coefficient. Using this calculation, a sample size of 74 patients was obtained.

Inclusion criteria included: individuals aged 18 years or older, a diagnosis of primary or secondary lung cancer (to maximize the target population given the high morbidity and mortality rates of lung cancer), and undergoing chemotherapy and/or radiotherapy for at least one month.

Exclusion criteria included: patients with severe communication and/or hearing deficits, those under palliative care, or those without sufficient cognitive ability to respond to the questionnaires, as assessed by the Mini-Mental State Examination (MMSE)^(15)^.

### Study Protocol

To collect sociodemographic and clinical data, a structured questionnaire was utilized, which was evaluated and validated by experts, including master’s and doctoral-level professionals in the field. This questionnaire provided information on the following variables: sex, age group, marital status, place of residence, occupation/profession, religion, family income, education level, race, time since diagnosis, type and duration of treatment, lifestyle habits, and personal and family history of cancer.

The facets of spirituality were assessed using the World Health Organization Quality of Life - Spirituality, Religiosity, and Personal Beliefs (WHOQOL-SRPB) instrument, developed by the World Health Organization (WHO) and validated in Brazil^(16)^. This instrument comprises 32 items divided into eight facets: connection to a spiritual being or force, meaning in life, admiration, wholeness and integration, spiritual strength, inner peace, hope, optimism, and faith. Responses are scored on a 5-point Likert scale, where “1 = nothing” and “5 = extremely.” Scores range from 0 to 20, with higher scores indicating better spiritual quality of life^(16)^.

Health-related quality of life (HRQoL) was assessed using the European Organization for Research and Treatment of Cancer Quality of Life Questionnaire (EORTC QLQ-C30), validated and adapted for the Brazilian population^(17)^. This questionnaire includes 30 items distributed across five functional scales (emotional, physical, cognitive, functional, and social performance), three symptom scales (fatigue, vomiting, nausea, and pain), and scales for global health and overall QoL. Scores range from 0 to 100, where 0 indicates the worst health status and 100 the best. Exceptions include the symptom scales, where higher scores represent more severe symptoms and lower QoL. Responses follow a 4-point Likert scale, where “1 = not at all” and “4 = very much^”(17)^.

### Data Analysis and Statistics

The collected data were stored in electronic spreadsheets using Microsoft Excel version 2019. The data were then organized, coded, and subsequently imported and processed using R software version 4.3.1. Analyses were conducted using descriptive and inferential statistics. For correlation analysis, Spearman’s correlation test was applied, as the variables were non-parametric. The magnitude of correlations was classified as follows: weak, if |r| < 0.3; moderate, if 0.3 ≤ |r| < 0.7; and strong, if |r| ≥ 0.7 ^(18)^.

## RESULTS

Among the participants, a higher frequency of females was observed (53%), with a mean age of 60 years. Participants were predominantly from other municipalities in Paraíba and João Pessoa (53% and 45%, respectively), married (53%), of mixed-race/mulatto ethnicity (60%), with an average income of 1.74 minimum wages and an average of 7.14 years of education. They predominantly identified as Catholic (57%) or Evangelical (26%). Regarding the WHOQOL-SRPB, the highest mean spirituality score was observed in the *faith* facet, with a score of 19, and in total spirituality, with a mean score of 17.3 (Table 1).

**Table 1:** Spirituality of lung cancer patients undergoing oncological treatment (N = 74). João Pessoa, Paraíba, Brazil, 2023

Variable	Mean ± SD
Faith	19 (6)
Connection	18 (2)
Hope	17.96 (2.27)
Strength	17 (2)
Peace	16.99 (2.37)
Meaning	16.55 (2.44)
Wholeness	16.5(2.2)
Admiration	16 (3)
Total Spirituality	17.3 (2.1)

In the EORTC QLQ-C30, the highest mean scores for symptoms were observed in: constipation (80.2), financial difficulties (66.2), fatigue (63), pain (53.6), loss of appetite (53.1), insomnia (49), and dyspnea (46). Regarding the functional scale, the highest mean scores were: cognitive function (71.2), emotional function (58), physical function (35), and social function (31.3). The global health scale presented the highest overall result among the scales, with a mean score of 61.15 (Table 2).

**Table 2:** Health-related quality of life of lung cancer patients undergoing oncological treatment (N = 74). João Pessoa, Paraíba, Brazil, 2023

Variable	Mean ± SD
Symptom Scale	50 (19)
Constipation	80.2 (32.6)
Financial difficulties	66.2 (32.9)
Fatigue	63 (28)
Pain	53.6 (36.3)
Loss of appetite	53.1 (38.2)
Insomnia	49 (39)
Dyspnea	46 (35.6)
Nausea and vomiting	27.2 (37.9)
Diarrhea	13.5 (30.2)
Functional Scale	43 (19)
Cognitive	71.2 (28.4)
Emotional	58 (28)
Physical functions	35 (25)
Social	31.3 (32)
Functional	18.5 (29.5)
Global Health Scale	61.15 (22)

When evaluating the relationship between spirituality and health-related quality of life (HRQoL), a weak positive correlation was observed between the *connection* score and financial difficulties (r = 0.29; p = 0.012), as well as with the *social* score (r = 0.27; p = 0.028). Similarly, a weak positive correlation was identified between the *meaning* score and the global health scale (r = 0.25; p = 0.041). Regarding the *admiration* score, a weak negative correlation was observed with the *functional* score (r = -0.24; p = 0.044). Additionally, a weak negative correlation was identified between insomnia and the *wholeness* score (r = -0.27; p = 0.024) (Table 3 and Table 4).

**Table 3:** Correlation between spirituality and health-related quality of life in lung cancer patients undergoing oncological treatment (N = 74). João Pessoa, Paraíba, Brazil, 2023

	Connection		Meaning		Admiration		Wholeness		Strength		Peace		Hope		Faith	
	**R**	**value *p***	**r**	**value *p***	**r**	**value *p***	**r**	**value *p***	**r**	**value *p***	**r**	**value *p***	**r**	**value *p***	**r**	**value *p***
Symptom Scale	0.12	0.31	-0.06	0.64	0.01	0.93	-0.11	0.37	0.06	0.65	-0.01	0.91	-0.03	0.78	-0.04	0.76
Fatigue	0.14	0.22	-0.05	0.67	-0.04	0.72	-0.04	0.75	0.13	0.28	-0.02	0.86	0.05	0.68	0.18	0.13
Nausea and vomiting	0.04	0.74	-0.06	0.65	0.14	0.25	-0.04	0.77	0.08	0.53	-0.02	0.86	-0.08	0.53	-0.04	0.78
Pain	0.1	0.4	-0.08	0.53	-0.19	0.13	-0.11	0.39	0.06	0.65	-0.04	0.75	-0.01	0.91	-0.11	0.37
Dyspnea	-0.01	0.94	0.03	0.81	0	0.99	-0.08	0.52	-0.08	0.53	-0.06	0.61	-0.13	0.28	-0.15	0.23
Insomnia	-0.16	0.17	-0.11	0.36	-0.12	0.33	-0.27	0.024^*^	-0.25	0.037^*^	-0.15	0.21	-0.18	0.15	-0.22	0.07
Loss of appetite	0.12	0.32	0.07	0.54	0.23	0.06	0.11	0.36	0.24	0.044^*^	0.19	0.12	0.13	0.3	0	0.99
Constipation	0.18	0.12	-0.09	0.45	-0.19	0.12	-0.12	0.33	0.11	0.36	0.03	0.81	0.01	0.95	0.1	0.43
Diarrhea	-0.07	0.56	-0.2	0.11	0.02	0.87	-0.15	0.23	-0.1	0.43	-0.13	0.3	-0.23	0.06	-0.08	0.51
Financial difficulties	0.29	0.012^*^	0.15	0.22	0.2	0.09	0.14	0.26	0.12	0.33	0.12	0.34	0.26	0.036^*^	0.21	0.08

* *Spearman’s linear correlation test.*

**Table 4:** Correlation between spirituality and health-related quality of life in lung cancer patients undergoing oncological treatment (N = 74). João Pessoa, Paraíba, Brazil, 2023

	Connection		Meaning		Admiration		Wholeness		Strength		Peace		Hope		Faith	
	**R**	**value *p***	**r**	**value *p***	**r**	**value *p***	**r**	**value *p***	**r**	**value *p***	**r**	**value *p***	**r**	**value *p***	**r**	**value *p***
Functional Scale	0.07	0.57	-0.17	0.17	-0.19	0.12	-0.23	0.06	-0.02	0.88	-0.11	0.36	-0.16	0.19	0	0.97
Physical functions	0.02	0.85	-0.01	0.93	-0.09	0.46	-0.13	0.3	0.05	0.69	0.01	0.94	-0.08	0.5	0.05	0.71
Functional	0.06	0.63	-0.17	0.17	-0.24	0.044^*^	-0.18	0.13	-0.01	0.91	-0.06	0.65	-0.01	0.96	0.07	0.56
Emotional	-0.03	0.79	-0.22	0.07	-0.15	0.23	-0.22	0.08	-0.09	0.45	-0.14	0.27	-0.22	0.07	-0.19	0.13
Cognitive	-0.17	0.16	-0.14	0.25	-0.16	0.2	-0.28	0.02	-0.19	0.12	-0.31	0.011^*^	-0.42	0.001^*^	-0.11	0.36
Social	0.27	0.028^*^	-0.02	0.87	0	0.99	0	0.98	0.17	0.17	0.09	0.45	0.15	0.23	0.15	0.22
Global Health Scale	0.18	0.15	0.25	0.041^*^	0.2	0.1	0.15	0.24	0.17	0.17	0.27	0.03	0.22	0.08	0.09	0.47

* *Spearman’s linear correlation test.*

Regarding *strength*, a weak negative correlation was observed with insomnia (r = -0.25; p = 0.037) and a weak positive correlation with loss of appetite (r = 0.24; p = 0.044). A weak negative correlation was identified between the *cognitive* score and *peace* (r = -0.31; p = 0.011). In relation to *hope*, a weak positive correlation was found with financial difficulties (r = 0.26; p = 0.036), along with a weak to moderate negative correlation with the *cognitive* score (r = -0.42; p = 0.001) (Table 3 and Table 4).

## DISCUSSION

There was a predominance of female participants, corroborating the increased incidence of lung cancer in women due to changes in smoking habits and their associated effects. This finding aligns with a prospective cohort study conducted in the United States among lung cancer patients^(19)^.

The participants’ mean age was 60 years. The literature indicates that lung cancer occurs more frequently in the elderly population, particularly among smokers and individuals exposed to biomass smoke, which causes long-term damage to pulmonary cells^(20)^. In Turkey, a descriptive and cross-sectional study involving lung cancer patients undergoing chemotherapy reported a general mean age of 61.43 ± 8.24 years^(21)^.

Regarding place of residence, some participants lived in João Pessoa, while most were from other municipalities, consistent with findings from other studies conducted in Brazil. These studies highlight that traveling from home to oncology reference centers is often associated with social inequalities and vulnerabilities, leading to significant financial costs and physical discomfort from long journeys. In Minas Gerais, patients’ place of residence even impacted the initiation of oncological treatment within 60 days of diagnosis, as recommended by current legislation^(4,22)^.

The sample showed a higher prevalence of married participants, a finding similar to those of studies conducted in Turkey and the Netherlands^(21,23)^. In these contexts, the spouse is the primary source of support and care for oncology patients, mitigating the negative effects of the disease and fostering positive responses to changes and adverse reactions during treatment^(24)^.

Concerning race/ethnicity, most participants self-identified as mixed-race/mulatto. It is worth noting that racial and ethnic characteristics in the context of lung cancer reflect increased exposure to risk factors due to social inequalities. A study conducted in Mato Grosso, a region in Brazil’s Midwest, found higher mortality rates among mixed-race individuals with lung cancer^(25)^.

The participants’ average income was 1.74 minimum wages, reflecting a low socioeconomic status. The financial repercussions of lung cancer have become a significant issue due to the added financial burden imposed by the disease, referred to as financial toxicity. This phenomenon directly impacts patients’ HRQoL^(26)^. In Paraná, an observational cross-sectional study concluded that lower financial difficulties were associated with higher HRQoL among participants^(26)^. In the United States, lung cancer patients living in areas with high poverty levels were more likely to experience delays in undergoing surgery, demonstrating that income level is associated with initiating treatment within the minimum recommended timeframe^(27)^.

Participants had an average of 7.14 years of education, indicating a low level of educational attainment. This finding aligns with a study conducted in the Netherlands, where many lung cancer patients exhibited low educational levels^(24)^. In Turkey, it was reported that only 46.7% of patients had completed elementary school^(21)^.

The majority of participants identified as Catholic or Evangelical. Similar findings were reported in the United States, where religious or spiritual practices were shown to serve as support mechanisms. Patients stated that trusting in a higher power or God represented a source of renewal when facing cancer^(28)^.

In the evaluation of spirituality, the highest mean score was observed in the *faith* facet, aligning with research conducted in Brazil, where patients presented a mean score of 18.56^(29)^. The positive effects of faith in lung cancer patients were highlighted in a study conducted in Texas, which demonstrated that *meaning/peace* and *faith* help manage patients’ and their spouses’ psychological symptoms both directly and indirectly, promoting mindfulness^(24)^.

For total spirituality, a mean score of 17.3 (on a scale of 0-20) was recorded, indicating high levels of spirituality. A study with similar results was conducted in Porto Alegre with women diagnosed with breast cancer, where the mean score was 17.76, using the WHOQOL-SRPB^(29)^. In other countries, such as South Korea and Turkey, lung cancer patients reported spirituality mean scores of 25.7 and 28.48 (on a scale of 0-48), respectively, based on the *Functional Assessment of Chronic Illness Therapy-Spiritual Well-Being Scale* (FACIT-sp)^(12,30)^. These disparities may be explained by cultural differences among countries.

In the evaluation of HRQoL, the highest mean scores in the symptom scale were observed for constipation, financial difficulties, fatigue, pain, loss of appetite, insomnia, and dyspnea. On the functional scale, the best mean scores were related to cognitive function, with the global health scale showing the highest mean score among all scales. These findings are similar to results found in Asia and South Korea, where the EORTC-QLQ-C30 instrument was also used^(11,12)^. This outcome may be associated with the positive effect of spiritual well-being on the QoL of lung cancer patients^(12)^.

Regarding the relationship between spirituality and HRQoL, a weak positive correlation was observed between the *connection* score and financial difficulties, as well as between *connection* and the social score of the functional scale. These findings suggest that the stronger the sense of connection, the better patients cope with financial difficulties and the higher their social performance. This effect may be explained by the positive influence of spirituality on life satisfaction, which fosters a less aggressive perception of the adverse effects of the disease^(30)^.

A weak positive correlation was also observed between the *meaning* score and the global health scale, indicating that as *meaning* increases, patients’ self-assessment of global health becomes more positive. It is inferred that stronger spiritual experiences are correlated with a greater sense of life purpose, life satisfaction, and the way individuals interpret their health condition, contributing to overall well-being^(8)^ and improved HRQoL.

In the *admiration* facet, a weak negative correlation was identified with the functional score, suggesting that higher levels of admiration are associated with lower functional performance. This may occur due to a decline in daily living activities, indicating impaired functionality in patients with lung cancer^(11)^. Additionally, it was observed that patients tend to dedicate more attention to spiritual matters, including admiration for a higher power.

A weak positive correlation was identified between insomnia and the *wholeness* score, which can be explained by the resilience patients develop when facing challenges such as insomnia, through an appreciation of life’s totality and nature. Regarding *strength*, a weak negative correlation was observed with insomnia, and a weak positive correlation was noted with loss of appetite. This suggests that greater spiritual strength is associated with fewer insomnia experiences, while loss of appetite increases in contrast to spiritual strength. These findings highlight a cause-and-effect relationship between spirituality and the physical symptoms associated with oncological diseases, as evidenced by studies conducted in Brazil and South Korea^(12,29)^.

A weak negative correlation was observed between the cognitive score and *peace*, indicating that as cognition declines, the perception of peace also tends to decrease. This diminished sense of peace in patients may be related to cognitive impairments caused by oncological treatment^(12)^, which hinder reflections and understanding of spiritual dimensions in individuals with lung cancer.

Regarding *hope*, a weak positive correlation was observed with financial difficulties. This finding suggests that as hope increases, the perception of financial challenges becomes more significant. A study conducted in Poland with breast and lung cancer patients indicated that hope is associated with a sense of life purpose, influencing how patients cope with the difficulties arising from the disease, including financial challenges^(8,26)^.

A weak to moderate negative correlation was also found between *hope* and the cognitive score, indicating that greater hope is associated with improved cognitive function. Hope serves as a facilitator for finding purpose and meaning in life, fostering optimism and happiness, which supports better cognitive performance in oncology patients^(8)^.

In this context, nurses should prioritize the patient-professional connection to promote health and comfort through spiritual care. This includes incorporating spiritual interventions into care plans, emphasizing the uniqueness of the patient and their family. Moreover, the importance of continuing education in healthcare institutions is underscored, preparing nursing professionals to manage spiritual care through practices such as mindfulness and music therapy. These efforts should involve multidisciplinary healthcare teams, aiming for a holistic approach to human care^(13,31)^.

### Study Limitations

The limitations of this study include the difficulty in comparing results with other studies that used different instruments to investigate the construct. It is suggested that future research with lung cancer patients undergoing oncological treatment use the same instruments to enable better correlations and comparisons between results.

### Contributions to Nursing, Health, or Public Policy

In the context of nursing practice, the data from this study provide important insights for care management, considering the spiritual dimension and its impact on HRQoL. These findings support humanized and holistic care within a multidisciplinary framework while also contributing to the advancement of research in this specific population by verifying the correlations between spirituality and HRQoL.

## CONCLUSIONS

Patients with lung cancer exhibited a high mean total spirituality score, with the faith facet standing out as the highest-scoring component. In HRQoL, high means were observed in the global health and functional scales. However, the highest means in the symptom scale were recorded in the domains of constipation, financial difficulties, fatigue, pain, loss of appetite, insomnia, and dyspnea, indicating poorer HRQoL related to symptoms.

Regarding correlations between scales, a weak positive correlation was found between the connection score and financial difficulties, as well as with the social score. A weak positive correlation was also observed between the meaning score and the global health scale. The admiration facet showed a weak negative correlation with the functional score, while insomnia exhibited a weak positive correlation with the wholeness score.

For the strength facet, there was a weak negative correlation with insomnia and a weak positive correlation with loss of appetite. Additionally, a weak negative correlation was observed between the cognitive score and peace. Finally, the hope facet showed a weak positive correlation with financial difficulties and a weak to moderate negative correlation with the cognitive score.

This study identifies that the facets of spirituality positively contribute to the HRQoL of lung cancer patients, encouraging reflection on the subject through a spiritual context approach. This approach promotes positive coping strategies and the development of resilience among patients and their families.
