# Supplementary material for: Spirituality and health-related quality of life in patients with lung cancer
Source: Rev Bras Enferm. 2025 Jul 11;78(3):e20240457. doi: 10.1590/0034-7167-2024-0457 (PMC12252921; doi:10.1590/0034-7167-2024-0457)
Supplement: Translation

# Translation: Espiritualidade e qualidade de vida relacionada à saúde de pacientes com câncer de pulmão

**DOI:** 10.1590/0034-7167-2024-0457pt

**Authors:** Erica Maria Belmiro dos Santos, Kátia Neyla de Freitas Macedo Costa, Maria das Graças Melo Fernandes, Lia Raquel de Carvalho Viana, Cleane Rosa Ribeiro da Silva, Maria Cristina Lins Oliveira Frazão, Gerlania Rodrigues Salviano Ferreira, Ana Luísa Fernandes Vieira Melo

**Affiliations:** I Universidade Federal da Paraíba. João Pessoa, Paraíba, Brasil

**Keywords:** Espiritualidade, Qualidade de Vida, Câncer de Pulmão, Enfermagem Oncológica, Atenção Integral à, Saúde

## Abstract

**Objetivo:**

Correlacionar a espiritualidade e a qualidade de vida relacionada à saúde de pacientes com câncer de pulmão.

**Métodos:**

Estudo com abordagem quantitativa, realizado com 74 pacientes em um hospital oncológico em João Pessoa, Paraíba, no período de julho a dezembro de 2023. As entrevistas foram conduzidas utilizando os instrumentos *World Health Organization Quality of Life Spirituality - Religiosity - Personal Beliefs* (WHOQOL-SRPB) e *European Organization for Research and Treatment of Cancer Care Quality of Life Questionnaire* (EORTC QLQ-C30). Os dados foram processados e analisados por meio de estatística descritiva e inferencial.

**Resultados:**

Observou-se uma correlação positiva fraca entre o escore de conexão e dificuldade financeira (p = 0,012) e uma correlação negativa fraca a moderada entre esperança e o escore cognitivo (p = 0,001) nas escalas de espiritualidade e qualidade de vida relacionada à saúde.

**Conclusão:**

Facetas da espiritualidade contribuem para a melhora da qualidade de vida relacionada à saúde dos pacientes, fornecendo subsídios para a concepção de um plano de cuidado individualizado.

## INTRODUÇÃO

Os casos de câncer de pulmão estão crescendo em nível global^(1)^. Nos Estados Unidos, é o segundo mais comum, sendo a principal causa de morte. Nos países em desenvolvimento, as taxas de incidência e prevalência continuam crescendo em consonância com o tabagismo, que é o principal fator de risco^(2)^.

No Brasil, estima-se 32 mil casos novos de câncer de pulmão para o triênio 2023-2025, de acordo com os dados do Instituto Nacional de Câncer (INCA)^(3)^, sendo classificado em pequenas células e de células não pequenas para fins terapêuticos e prognósticos. Ambos detêm alta letalidade, baixa taxa de cura e sobrevida^(4)^.

O câncer de pulmão e seu tratamento estão relacionados a sintomas incapacitantes, como dor, dispneia, tosse e fadiga, que, dentre outros fatores estressantes, podem apresentar várias respostas cognitivas e afetivas, desencadeando sintomas depressivos, ansiedade e sentimentos negativos em relação a Deus pelo acometimento da patologia. Essas manifestações psicoespirituais interferem na adesão ao tratamento e na qualidade de vida (QV)^(5)^.

Em contrapartida, pesquisadores identificaram que o bem-estar espiritual desses pacientes representa um fator de proteção contra o sofrimento causado pela doença, o que contribui para uma melhor QV geral^(6-8)^. A espiritualidade é considerada um aspecto indissociável da subjetividade humana, por influenciar no bem-estar físico, psicossocial e cultural. Configura-se como a busca individual por significado, sentido para viver e relação com o sagrado^(9)^. Desse modo, as facetas espirituais contemplam a multidimensionalidade da pessoa e estão interligadas ao seu bem-estar e à qualidade de vida relacionada à saúde (QVRS).

Segundo a Organização Mundial da Saúde (OMS), a QV é definida como a percepção que o indivíduo tem de si mesmo, considerando o meio em que vive^(10)^. Quando se relaciona a intervenções de saúde, denomina-se QVRS, sendo um construto que considera aspectos cognitivos, funcionais, psicológicos e sociais do indivíduo^(10,11)^. No contexto do câncer de pulmão, a QVRS é afetada por diversos fatores relacionados à doença oncológica, que podem acarretar problemas físicos, emocionais, familiares e sociais^(11)^.

Estudo realizado em Singapura, na Ásia, em pacientes com câncer de pulmão apontou que maiores níveis de necessidades psicológicas e no estado funcional estiveram associados a pior QVRS^(11)^. Nesse ínterim, a espiritualidade pode atenuar o comprometimento da QVRS por meio de estratégias que permitam ao paciente ressignificar o sentido da vida, promovendo resiliência e determinação frente às dificuldades impostas pela patologia^(12)^.

Frente a este cenário, a dimensão espiritual deve ser incluída na assistência de enfermagem por meio do plano de cuidados, instrumentalizando ações que estimulem o desenvolvimento da crença dos pacientes como meio de fortalecimento, para assim promover apoio e conforto durante a vivência da doença oncológica^(13)^. A hipótese deste estudo é que há influência da espiritualidade na QVRS de pacientes com câncer de pulmão. Assim, a correlação entre estas duas variáveis destaca a relevância da multidimensionalidade da pessoa com câncer, auxiliando na prevenção dos impactos negativos ocasionados pela patologia.

## OBJETIVO

Correlacionar a espiritualidade e a QVRS de pacientes com câncer de pulmão em tratamento oncológico.

## MÉTODOS

### Aspectos éticos

O projeto foi aprovado pelo Comitê de Ética em Pesquisa (CEP) do Centro de Ciências da Saúde (CCS) da Universidade Federal da Paraíba (UFPB). O estudo foi desenvolvido de acordo com o recomendado pela Resolução n° 466/2012 do Conselho Nacional de Saúde (CNS) do Ministério da Saúde (MS). Todos os participantes assinaram, na ocasião da coleta de dados, o Termo de Consentimento Livre e Esclarecido (TCLE). Foram convidados a participar aqueles que atenderam aos critérios de inclusão, sendo informados sobre os riscos e benefícios, bem como sobre a preservação do anonimato em todas as etapas da pesquisa.

### Desenho, período e local de estudo

Estudo descritivo e transversal, com abordagem quantitativa^(14)^, norteado pela ferramenta *Strengthening the Reporting of Observational Studies in Epidemiology* (STROBE). Foi realizado em um hospital de referência estadual no tratamento oncológico, de grande porte e alta complexidade, localizado no município de João Pessoa, estado da Paraíba, Brasil. A coleta de dados ocorreu por meio de entrevistas individuais realizadas na sala de espera para a sessão de quimioterapia ou radioterapia, no período de julho a dezembro de 2023.

### População e amostra; Critérios de inclusão e exclusão

A seleção da amostra foi baseado no quantitativo de pacientes atendidos pelo cirurgião torácico no ambulatório do serviço de referência, de janeiro a junho de 2023 que totalizou 253 pacientes, a partir do pressuposto que a população é homogênea em relação as principais variáveis do estudo, foi considerado pelo cálculo amostral um tamanho de amostra capaz de detectar correlações fracas, moderadas ou fortes com coeficiente de correlação linear mínimo de 0,32, o nível de significância foi de 5%, ou seja, confiança de 95% e o poder de 80%. Calculando a amostra com a seguinte expressão:


$$n=\left(\frac{Z_{a}+Z_{\beta }}{\frac{1}{2}\log\left(\frac{1+r}{1-r}\right)}\right)+3,$$

Em que α é o nível de significância, 1 - *β* é o poder, *Z* ( . ) é o quantil da distribuição normal padrão e r é a estimativa do coeficiente de correlação linear, desta forma obtendo-se uma amostra de 74 pacientes.

Os critérios de inclusão foram: idade maior ou igual a 18anos, ter diagnóstico de câncer de pulmão primário ou secundário para abranger ao máximo o público-alvo, uma vez que o câncer de pulmão detém altas taxas de morbimortalidade, bem como está em tratamento quimioterápico e/ou radioterápico há no mínimo um mês. Critérios para exclusão foram: pacientes com déficit graves de comunicação e/ou audição, está em cuidados paliativos e não possuir condição cognitiva para responder os questionários, verificado através do Mini Exame do Estado Mental (MEEM)^(15)^.

### Protocolo do estudo

Para a obtenção dos dados sociodemográficos e clínicos, foi utilizado um questionário estruturado, avaliado e validado por juízes, mestres e doutores da área. Este questionário apresenta informações sobre as variáveis: sexo, faixa etária, estado civil, procedência, ocupação/profissão, religião, renda familiar, escolaridade, raça, tempo de diagnóstico, tipo e tempo de tratamento, hábitos de vida, histórico individual e familiar para o câncer.

As facetas da espiritualidade foram verificadas por meio do instrumento *World Health Organization Quality of Life - Espiritualidade, Religiosidade e Crenças Pessoais* (WHOQOL-SRPB), da OMS, validado no Brasil^(16)^. O instrumento possui 32 itens divididos em oito facetas: conexão a um ser ou força espiritual, sentido na vida, admiração, totalidade e integração, força espiritual, paz interior, esperança, otimismo e fé. As respostas seguem uma escala de Likert de 5 pontos, em que “1 = nada” e “5 = extremamente”. Os escores variam de 0 a 20, sendo que escores mais elevados indicam melhor QV espiritual^(16)^.

A QV relacionada à saúde foi avaliada pelo *European Organization for Research and Treatment of Cancer Quality of Life Questionnaire* (EORTC QLQ-C30), validado e adaptado à população brasileira^(17)^. O questionário é composto por 30 itens distribuídos em cinco escalas funcionais (desempenho emocional, físico, cognitivo, funcional e social), três escalas de sintomas (fadiga, vômito, náusea e dor) e escalas de QV e estado de saúde global. O escore varia de 0 a 100, em que 0 indica pior estado de saúde e 100 melhor estado de saúde, com exceção das escalas de sintomas, onde escores mais altos representam mais sintomas e pior QV. As respostas seguem uma escala de Likert de 4 pontos, em que “1 = não” e “4 = muito” ^(17)^.

### Análise dos resultados e estatística

Os dados obtidos foram armazenados em planilhas eletrônicas no programa Microsoft Excel versão 2019, organizados, codificados e, posteriormente, importados e processados pelo software R versão 4.3.1. As análises foram realizadas por meio de estatística descritiva e inferencial. Para a correlação, foi aplicado o Teste de Correlação de Spearman, uma vez que se tratam de variáveis não paramétricas. A classificação da magnitude das correlações foi definida como: fraca, se |r| < 0,3; moderada, se 0,3 ≤ |r| < 0,7; e forte, se |r| ≥ 0,7 ^(18)^.

## RESULTADOS

Dentre os participantes, foi observada uma maior frequência do sexo feminino (53%), com média de idade de 60 anos, provenientes de outras cidades paraibanas e de João Pessoa (53% e 45%, respectivamente), casados (53%), de raça parda/mulata (60%), com renda média de 1,74 saláriocmínimo, média de 7,14 anos de estudo, e pertencentes às religiões católica e evangélica (57% e 26%, respectivamente). Em relação ao WHOQOL-SRPB, foi possível observar a maior média de espiritualidade na faceta fé, com escore 19, e na espiritualidade total, com média de 17,3 (Tabela 1).

**Tabela 1:** Espiritualidade de pacientes com câncer de pulmão em tratamento oncológico (N=74). João Pessoa, Paraíba, Brasil 2023

Variável	Média ± DP
Fé	19 (6)
Conexão	18 (2)
Esperança	17,96 (2,27)
Força	17 (2)
Paz	16,99 (2,37)
Sentido	16,55 (2,44)
Inteireza	16,5(2,2)
Admiração	16 (3)
Espiritualidade total	17,3 (2,1)

Na EORTC-QLQ-C30, verificou-se que as maiores médias nos sintomas foram: constipação (80,2), dificuldade financeira (66,2), fadiga (63), dor (53,6), perda do apetite (53,1), insônia (49) e dispneia (46). Quanto à escala funcional, as maiores médias foram: função cognitiva (71,2), função emocional (58), funções físicas (35) e função social (31,3). A escala de saúde global apresentou o maior resultado entre as escalas, com média de 61,15 (Tabela 2).

**Tabela 2:** Qualidade de vida relacionada à saúde de pacientes com câncer de pulmão em tratamento oncológico (N=74). João Pessoa, Paraíba, Brasil 2023

Variável	Média ± DP
Escala de sintomas	50 (19)
Constipação	80,2 (32,6)
Dificuldade financeira	66,2 (32,9)
Fadiga	63 (28)
Dor	53,6 (36,3)
Perda de apetite	53,1 (38,2)
Insônia	49 (39)
Dispneia	46 (35,6)
Náuseas e vômitos	27,2 (37,9)
Diarreia	13,5 (30,2)
Escala funcional	43 (19)
Cognitiva	71,2 (28,4)
Emocional	58 (28)
Funções físicas	35 (25)
Social	31,3 (32)
Funcional	18,5 (29,5)
Escala de saúde global	61,15 (22)

Ao avaliar a relação entre espiritualidade e a QV relacionada à saúde, observou-se uma correlação positiva fraca entre o escore *conexão* e *dificuldade financeira* (r = 0,29; p = 0,012), bem como com o escore *social* (r = 0,27; p = 0,028). Da mesma forma, identificou-se uma correlação positiva fraca entre o escore *sentido* e a escala de saúde global (r = 0,25; p = 0,041). Em relação ao escore *admiração*, observou-se uma correlação negativa fraca com o escore funcional (r = -0,24; p = 0,044). Além disso, foi identificada uma correlação negativa fraca entre *insônia* e o escore *inteireza* (r = -0,27; p = 0,024) (Tabela 3 e Tabela 4).

**Tabela 3:** Correlação entre a espiritualidade e a qualidade de vida relacionada à saúde de pacientes com câncer de pulmão em tratamento oncológico (N=74). João Pessoa, Paraíba, Brasil 2023

	Conexão		Sentido		Admiração		Inteireza		Força		Paz		Esperança		Fé	
	**R**	**valor de *p***	**r**	**valor de *p***	**r**	**valor de *p***	**r**	**valor de *p***	**r**	**valor de *p***	**r**	**valor de *p***	**r**	**valor de *p***	**r**	**valor de *p***
Escala de sintomas	0,12	0,31	-0,06	0,64	0,01	0,93	-0,11	0,37	0,06	0,65	-0,01	0,91	-0,03	0,78	-0,04	0,76
Fadiga	0,14	0,22	-0,05	0,67	-0,04	0,72	-0,04	0,75	0,13	0,28	-0,02	0,86	0,05	0,68	0,18	0,13
Náuseas e vômitos	0,04	0,74	-0,06	0,65	0,14	0,25	-0,04	0,77	0,08	0,53	-0,02	0,86	-0,08	0,53	-0,04	0,78
Dor	0,1	0,4	-0,08	0,53	-0,19	0,13	-0,11	0,39	0,06	0,65	-0,04	0,75	-0,01	0,91	-0,11	0,37
Dispneia	-0,01	0,94	0,03	0,81	0	0,99	-0,08	0,52	-0,08	0,53	-0,06	0,61	-0,13	0,28	-0,15	0,23
Insônia	-0,16	0,17	-0,11	0,36	-0,12	0,33	-0,27	0,024^*^	-0,25	0,037^*^	-0,15	0,21	-0,18	0,15	-0,22	0,07
Perda de apetite	0,12	0,32	0,07	0,54	0,23	0,06	0,11	0,36	0,24	0,044^*^	0,19	0,12	0,13	0,3	0	0,99
Constipação	0,18	0,12	-0,09	0,45	-0,19	0,12	-0,12	0,33	0,11	0,36	0,03	0,81	0,01	0,95	0,1	0,43
Diarreia	-0,07	0,56	-0,2	0,11	0,02	0,87	-0,15	0,23	-0,1	0,43	-0,13	0,3	-0,23	0,06	-0,08	0,51
Dificuldade financeira	0,29	0,012^*^	0,15	0,22	0,2	0,09	0,14	0,26	0,12	0,33	0,12	0,34	0,26	0,036^*^	0,21	0,08

* *Teste correlação linear de Spearman*

**Tabela 4:** Correlação entre a espiritualidade e a qualidade de vida relacionada à saúde de pacientes com câncer de pulmão em tratamento oncológico (N=74). João Pessoa, Paraíba, Brasil 2023

	Conexão		Sentido		Admiração		Inteireza		Força		Paz		Esperança		Fé	
	**R**	**valor de *p***	**r**	**valor de *p***	**r**	**valor de *p***	**r**	**valor de *p***	**r**	**valor de *p***	**r**	**valor de *p***	**r**	**valor de *p***	**r**	**valor de *p***
Escala funcional	0,07	0,57	-0,17	0,17	-0,19	0,12	-0,23	0,06	-0,02	0,88	-0,11	0,36	-0,16	0,19	0	0,97
Funções físicas	0,02	0,85	-0,01	0,93	-0,09	0,46	-0,13	0,3	0,05	0,69	0,01	0,94	-0,08	0,5	0,05	0,71
Funcional	0,06	0,63	-0,17	0,17	-0,24	0,044^*^	-0,18	0,13	-0,01	0,91	-0,06	0,65	-0,01	0,96	0,07	0,56
Emocional	-0,03	0,79	-0,22	0,07	-0,15	0,23	-0,22	0,08	-0,09	0,45	-0,14	0,27	-0,22	0,07	-0,19	0,13
Cognitiva	-0,17	0,16	-0,14	0,25	-0,16	0,2	-0,28	0,02	-0,19	0,12	-0,31	0,011^*^	-0,42	0,001^*^	-0,11	0,36
Social	0,27	0,028^*^	-0,02	0,87	0	0,99	0	0,98	0,17	0,17	0,09	0,45	0,15	0,23	0,15	0,22
Escala de saúde global	0,18	0,15	0,25	0,041^*^	0,2	0,1	0,15	0,24	0,17	0,17	0,27	0,03	0,22	0,08	0,09	0,47

* *Teste correlação linear de Spearman*

No que tange à força, observou-se uma correlação negativa fraca com *insônia* (r = -0,25; p = 0,037) e uma correlação positiva fraca com *perda de apetite* (r = 0,24; p = 0,044). Identificou-se uma correlação negativa fraca entre o escore *cognitivo* e *paz* (r = -0,31; p = 0,011). Em relação à *esperança*, houve uma correlação positiva fraca com *dificuldade financeira* (r = 0,26; p = 0,036) e uma correlação negativa fraca a moderada com o escore *cognitivo* (r = -0,42; p = 0,001) (Tabela 3 e Tabela 4).

## DISCUSSÃO

Em relação ao perfil sociodemográfico, houve predominância do sexo feminino, o que ratifica o aumento da incidência do câncer de pulmão nas mulheres devido à mudança nos hábitos relacionados ao tabagismo e seus efeitos. Este achado é semelhante ao de um estudo de coorte prospectiva realizado nos Estados Unidos com pacientes com câncer de pulmão^(19)^.

A idade média dos participantes foi de 60 anos. A literatura aponta que o câncer de pulmão acomete com maior frequência a população idosa, especialmente entre fumantes e aqueles expostos à fumaça de biomassa, que causa danos às células pulmonares a longo prazo^(20)^. Na Turquia, uma pesquisa descritiva e transversal com pacientes com câncer de pulmão em tratamento quimioterápico identificou uma média geral de idade de 61,43 ± 8,24 anos^(21)^.

No que concerne à procedência, uma parte dos participantes residia em João Pessoa, enquanto a maioria era proveniente de outras cidades, corroborando pesquisas realizadas no cenário brasileiro. Essas pesquisas apontam que o deslocamento da residência até os serviços de saúde de referência em oncologia está relacionado a desigualdades e vulnerabilidades sociais, gerando gastos econômicos exacerbados e desconforto físico devido às longas viagens. Em Minas Gerais, a residência dos pacientes interferia até no início do tratamento oncológico antes de 60 dias após o diagnóstico, conforme recomendado pela legislação vigente^(4,22)^.

A amostra apresentou maior prevalência de entrevistados casados, achado semelhante aos de estudos realizados na Turquia e na Holanda^(21,23)^. Nesses contextos, o cônjuge é a principal fonte de apoio e cuidado para pacientes oncológicos, amenizando os efeitos negativos da doença e colaborando com respostas positivas frente às mudanças e reações adversas do tratamento^(24)^.

Quanto à cor/raça, a maioria dos participantes se autodeclarou parda/mulata. Vale salientar que características raciais e étnicas no contexto do câncer de pulmão representam uma maior exposição aos fatores de risco devido às desigualdades sociais. Uma pesquisa realizada no Mato Grosso, região Centro-Oeste do Brasil, identificou elevadas taxas de óbito entre pessoas pardas com câncer de pulmão^(25)^.

A renda média dos participantes foi de 1,74 salários mínimos, caracterizando um baixo nível socioeconômico. As repercussões negativas da baixa renda no câncer de pulmão tornaram-se uma problemática devido ao ônus financeiro acrescido pela patologia, o que é considerado um efeito adverso denominado *toxicidade financeira*. Esse fenômeno repercute diretamente na QVRS dos pacientes^(26)^. No Paraná, um estudo observacional e transversal concluiu que quanto menores as dificuldades financeiras, maior era a QVRS dos participantes^(26)^. Nos Estados Unidos, pacientes com câncer de pulmão residentes em áreas com alto índice de pobreza apresentavam maior probabilidade de atraso na realização de cirurgias, demonstrando que o nível de renda está associado ao início do tratamento dentro do prazo mínimo estabelecido^(27)^.

Observou-se uma média de 7,14 anos de estudo entre os participantes, caracterizando um baixo nível de escolaridade, o que é consistente com estudo realizado na Holanda, onde grande parte dos entrevistados com câncer de pulmão apresentavam baixa escolaridade^(24)^. Na Turquia, foi identificado que apenas 46,7% dos pacientes tinham o ensino fundamental completo^(21)^.

A maioria dos participantes se autodeclarou de religião católica ou evangélica. Dados semelhantes foram encontrados nos Estados Unidos, onde se evidenciou a prática religiosa/espiritual como um suporte. Pacientes declararam que confiar em um poder superior ou em Deus representava uma fonte de renovação no enfrentamento do câncer^(28)^.

Na avaliação da espiritualidade, obteve-se maior média na faceta *fé*, corroborando pesquisa desenvolvida no Brasil, onde os pacientes apresentaram média de 18,56^(29)^. Os efeitos positivos da fé em pacientes com câncer de pulmão foram observados em estudo realizado no Texas, que mostrou que o significado/paz e a fé ajudam a gerenciar, direta e indiretamente, os sintomas psicológicos dos pacientes e de seus cônjuges, promovendo atenção plena^(24)^.

Quanto à espiritualidade total, constatou-se uma média de 17,3 (escore de 0-20), indicando altos níveis de espiritualidade. Uma pesquisa com resultados semelhantes foi realizada em Porto Alegre com mulheres com câncer de mama, onde a média foi 17,76, utilizando o WHOQOL-SRPB^(29)^. Em outros países, como Coreia do Sul e Turquia, pacientes com câncer de pulmão apresentaram médias de espiritualidade de 25,7 e 28,48 (escore de 0-48), respectivamente, de acordo com a escala *Functional Assessment of Chronic Illness Therapy-Spiritual Well-Being Scale* (FACIT-sp)^(12,30)^. Essa disparidade pode ser explicada pelas diferenças culturais entre os países.

Na avaliação da QVRS, as maiores médias na escala de sintomas foram observadas em constipação, dificuldade financeira, fadiga, dor, perda de apetite, insônia e dispneia. Na escala funcional, as melhores médias foram relacionadas à função cognitiva, com destaque para a escala de saúde global, que apresentou a maior média entre as demais. Esses achados são semelhantes aos resultados encontrados na Ásia e na Coreia do Sul, onde também foi utilizado o instrumento EORTC-QLQ-C30^(11,12)^. Tal resultado pode estar associado ao efeito positivo do bem-estar espiritual na QV dos pacientes com câncer de pulmão^(12)^.

No que concerne à relação entre espiritualidade e QVRS, foi observada uma correlação positiva fraca entre o escore *conexão* e *dificuldade financeira*, assim como entre *conexão* e o escore *social* da escala funcional. Esses achados evidenciam que, quanto maior a conexão, melhor os pacientes lidam com as dificuldades financeiras e maior é o seu desempenho social. Esse efeito pode ser explicado pela influência positiva da espiritualidade na satisfação com a vida, proporcionando uma percepção menos agressiva dos efeitos adversos da doença^(30)^.

Observou-se também uma correlação fraca positiva entre *sentido* e a escala de saúde global, indicando que, à medida que o *sentido* aumenta, mais positiva é a autoavaliação da saúde global. Infere-se que maiores experiências espirituais estão correlacionadas com o sentido da vida, satisfação com a vida e a forma como as pessoas interpretam sua condição de saúde, contribuindo para o bem-estar geral^(8)^ e uma melhor QVRS.

Na faceta *admiração*, identificou-se uma correlação negativa fraca com o escore funcional, sugerindo que, quanto maiores os níveis de *admiração*, menor é o desempenho funcional. Isso pode ocorrer devido ao declínio das atividades de vida diária como sinais de comprometimento da funcionalidade de pacientes com câncer de pulmão^(11)^. Além disso, observa-se uma maior dedicação dos pacientes às questões espirituais, incluindo a *admiração* por um ser superior.

Identificou-se uma correlação positiva fraca entre *insônia* e o escore *inteireza*, justificando-se pela capacidade de resiliência adquirida pelos pacientes diante de desafios como a insônia, por meio da apreciação da totalidade da vida e da natureza. No que tange à *força*, houve uma correlação negativa fraca com *insônia* e uma correlação positiva fraca com *perda de apetite*. Isso sugere que, quanto maior a *força espiritual*, menores são as experiências relacionadas à insônia, enquanto a *perda de apetite* aumenta em contrapartida à *força espiritual*. Observa-se, assim, uma relação de causa e efeito entre a espiritualidade e os sintomas físicos relacionados à doença oncológica, o que também foi evidenciado em estudos realizados no Brasil e na Coreia do Sul^(12,29)^.

Houve uma correlação negativa fraca entre o escore *cognitivo* e *paz*, indicando que, à medida que a cognição diminui, a percepção de paz também tende a diminuir. O comprometimento da percepção de paz por esses pacientes pode estar relacionado às alterações cognitivas causadas pelo tratamento oncológico^(12)^, o que impede reflexões e compreensões sobre a dimensão espiritual pela pessoa com câncer de pulmão.

Em relação à *esperança*, observou-se uma correlação positiva fraca com *dificuldade financeira*. Isso sugere que, quando a *esperança* aumenta, mais desafiadora se torna a percepção sobre a dificuldade financeira. Um estudo realizado na Polônia com pacientes com câncer de mama e pulmão indicou que a *esperança* está associada ao sentido na vida, influenciando a maneira como os pacientes enfrentam as dificuldades decorrentes da doença, incluindo as financeiras^(8,26)^.

Houve também uma correlação negativa fraca a moderada entre *esperança* e o escore *cognitivo*, indicando que, quanto maior a *esperança*, melhor é a função cognitiva. A *esperança* atua como facilitadora para encontrar propósito e significado na vida, promovendo otimismo e felicidade, o que corrobora para um melhor desempenho cognitivo de pacientes oncológicos^(8)^.

Nesse contexto, o enfermeiro deve ter como alicerce a conexão paciente-profissional para promover saúde e conforto por meio do cuidado espiritual, incluindo intervenções espirituais no plano de cuidado, valorizando a singularidade do paciente e de seus familiares. Ademais, destaca-se a importância da prática da educação permanente nas instituições, preparando os profissionais de enfermagem para a gestão do cuidado espiritual por meio de práticas como *mindfulness* e musicoterapia. Isso deve incluir interações com equipes multidisciplinares em saúde, visando uma abordagem holística do ser humano^(13,31)^.

### Limitações do Estudo

As limitações deste estudo incluem a dificuldade em comparar os resultados com outros estudos que utilizaram instrumentos diferentes para a investigação do construto. Sugere-se que novas pesquisas com pacientes com câncer de pulmão em tratamento oncológico utilizem os mesmos instrumentos, a fim de permitir melhores correlações e comparações entre os resultados obtidos.

### Contribuições para a área da Enfermagem, Saúde ou Política Pública

No contexto da prática de enfermagem, os dados deste estudo fornecem subsídios importantes para a gestão do cuidado, considerando a dimensão espiritual e seu impacto na QVRS. Eles dão suporte à assistência humanizada e holística no âmbito multiprofissional, além de contribuírem para o avanço das pesquisas nessa população específica, ao verificar as correlações existentes entre espiritualidade e QVRS.

## CONCLUSÕES

Os pacientes com câncer de pulmão apresentaram uma alta média de espiritualidade total, com destaque para a faceta *fé*, que obteve a maior média. Na QVRS, foram observadas médias altas na escala de saúde global e funcional. Contudo, na escala de sintomas, registraram-se as maiores médias nos domínios constipação, dificuldade financeira, fadiga, dor, perda de apetite, insônia e dispneia, indicando pior QVRS relacionada aos sintomas.

No que se refere às correlações entre as escalas, foi verificada uma correlação positiva fraca entre o escore *conexão* e *dificuldade financeira*, assim como com o escore *social*. Também foi observada uma correlação positiva fraca entre *sentido* e a escala de saúde global. A faceta *admiração* apresentou correlação negativa fraca com o escore funcional, enquanto *insônia* teve uma correlação positiva fraca com o escore *inteireza*.

Quanto à faceta *força*, houve uma correlação negativa fraca com *insônia* e uma correlação positiva fraca com *perda de apetite*. Observou-se ainda uma correlação negativa fraca entre o escore *cognitivo* e *paz*. Por fim, a faceta *esperança* apresentou uma correlação positiva fraca com *dificuldade financeira* e uma correlação negativa fraca a moderada com o escore *cognitivo*.

Dessa forma, esta pesquisa identifica que as facetas da espiritualidade contribuem positivamente para a QVRS dos pacientes com câncer de pulmão, conduzindo a uma reflexão sobre o tema por meio da abordagem do contexto espiritual. Tal abordagem promove o estímulo ao enfrentamento positivo e ao desenvolvimento da resiliência por parte dos pacientes e suas famílias.
